# Supplementary material for: Splice-Junction-Based Mapping of Alternative Isoforms in the Human Proteome
Source: Cell Rep. Author manuscript; Available in PMC 2020 Jan 15. (PMC6961840; doi:10.1016/j.celrep.2019.11.026)
Supplement: 2 [file NIHMS1546469-supplement-2.zip › DF1/PXD010154/Thyroid/Thyroid_1_TG_LQASGAGTWCVDPASGEELRPGSSSSAQCPR.pdf]

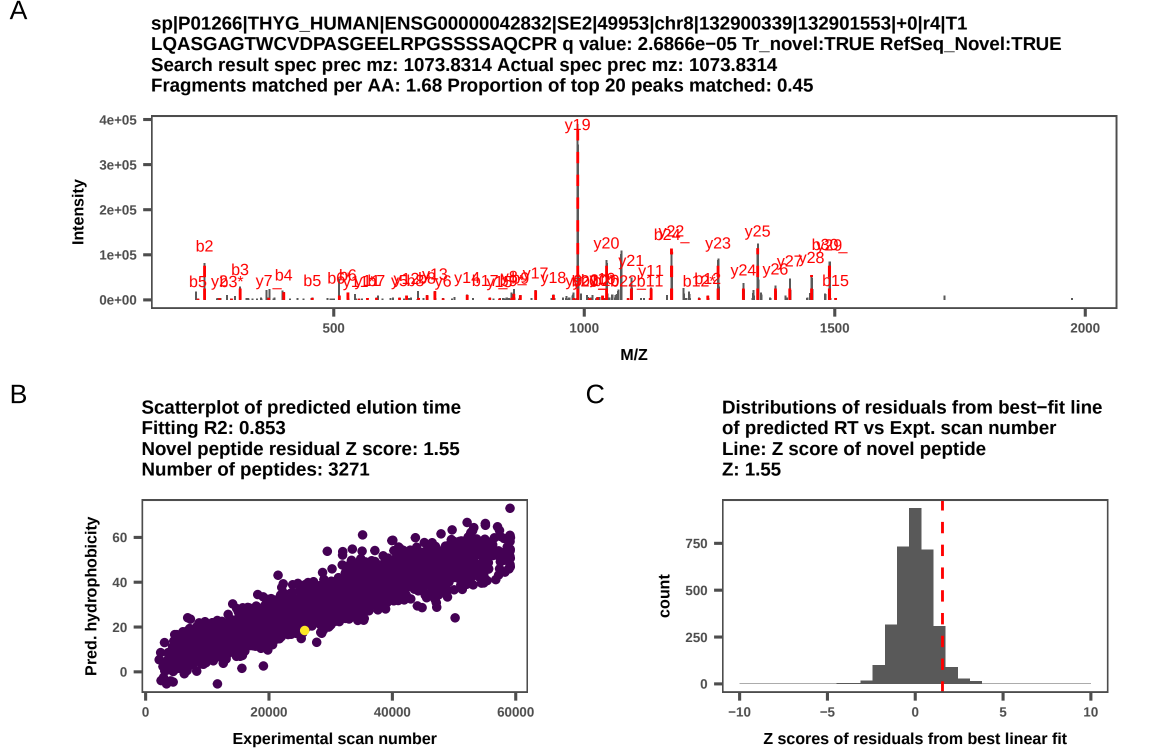
A
sp|P01266|THYG_HUMAN|ENSG00000042832|SE2|49953|chr8|132900339|132901553|+0|r4|T1
LQASGAGTWCVDPASGEELRPGSSSSAQCPR q value: 2.6866e−05 Tr_novel:TRUE RefSeq_Novel:TRUE
Search result spec prec mz: 1073.8314 Actual spec prec mz: 1073.8314
Fragments matched per AA: 1.68 Proportion of top 20 peaks matched: 0.45
Intensity
4e+05
3e+05
2e+05
1e+05
0e+00
M/Z
500
1000
1500
2000
y19
b2
b3
y20
y21
y23
y25
y24
y26
y28
b15
B
Scatterplot of predicted elution time
Fitting R2: 0.853
Novel peptide residual Z score: 1.55
Number of peptides: 3271
Pred. hydrophobicity
60
40
20
0
0
20000
40000
60000
Experimental scan number
C
Distributions of residuals from best−fit line
of predicted RT vs Expt. scan number
Line: Z score of novel peptide
Z: 1.55
count
750
500
250
0
−10
−5
0
5
10
Z scores of residuals from best linear fit
